# Updated Italian checklist of Soldier Flies (Diptera, Stratiomyidae)

**DOI:** 10.3897/zookeys.336.6016

**Published:** 2013-09-27

**Authors:** Franco Mason

**Affiliations:** 1 MiPAAF, Corpo Forestale dello Stato, CNBFVR, Centro Nazionale Biodiversità Forestale “Bosco Fontana“ di Verona, Via Carlo Ederle 16/a, I – 37100 Verona, Italy; 2 Consiglio Nazionale delle Ricerche, Istituto di Biologia Agroambientale e Forestale (CNR–IBAF). Via Salaria km 29,300 – 00015 Monterotondo (Rome, Italy)

**Keywords:** Italy, identification keys, faunistic, checklist, forest, Life Project

## Abstract

An updated checklist for Stratiomyidae of Italy is presented. Previous knowledge and information are put together in a comprehensive way, integrated also with results obtained by sampling with Malaise traps in some of the test areas of the LIFE+ project ManFor C.BD.

At the time of writing, with 91 known species, the Italian fauna of Stratiomyidae is the richest in Europe. *Neopachygaster meromelas* (Dufour, 1841) and *Zabrachia minutissima* (Zetterstedt, 1838) are new to the Italian fauna. A comprehensive key to the European species of *Chorisops* Rondani, 1856 is given.

## Introduction

In the recent decades, stimulated by the monograph of Rozkošný (1982, 1983), faunistic research on Stratiomyidae has received a remarkable stimulus throughout Europe. In Italy the latest studies are reported in the following contributions: Adamo (2008), Mason (1988a), Mason (1988b), Mason (1989), Biondi et al. (1991), Troiano (1995), Troiano and Toscano (1995), Mason and Rozkošný (2003), Mason (2003), Mason (2004), Mason (2005), Mason et al. (2006), Mason and Rozkošný (2008), Whitmore et al. (2008), Stuke (2008) and Mason et al. (2009). The faunistic data collected in this paper (see Appendix) are preliminary to the biodiversity studies in the framework of the project LIFE09 ENV/IT/000078 ManFor C.BD., “Managing forests for multiple purposes: carbon, biodiversity and socio–economic wellbeing” and were partly integrated by sampling with Malaise traps in some of the project test areas. The Italian species are listed in Table 1, according to the criteria of the “Checklist of the Italian Fauna” (Minelli et al. 1995; Mason and Krivosheina 1995). The identifications were made using Rozkošný (1982, 1983), Troiano (1995), Troiano and Toscano (1995) and Krivosheina and Rozkošný (1990). The nomenclature and the list of the species known to Italy follows “Fauna Europaea” (Rozkošný 2012). Abbreviations of the collections: FMCV (Franco Mason, Verona, Italy); MCSNG (Museo Civico di Storia Naturale, Genova, Italy) CNBFVR (Centro Nazionale Biodiversità Forestale “Bosco Fontana“ Verona, Italy).

**Table 1.** Updated Italian checklist of Stratiomyidae. Abbreviations: Italian administrative regions (cf. Minelli et al. 1995). N = Northern Italy: Em = Emilia-Romagna, FVG = Friuli-Venezia Giulia, Li = Liguria, Lo = Lombardy, Pi = Piedmont, TAA = Trentino-Alto Adige, V = Venetia, VA = Val d'Aosta. S = Peninisular Italy: Abr = Abruzzo, Ba = Basilicata, Ca = Calabria, Cp = Campania, La = Latium, Ma = Marches, Mo = Molise, Pu = Apulia, To = Tuscany, Um = Umbria. Si = Sicily and small circum-Sicilian islands, Sa = Sardinia and small circum-Sardinian islands.

**Taxa**		**N**								**S**										**Sa**	**Si**
		**Em**	**FVG**	**Li**	**Lo**	**Pi**	**TAA**	**VA**	**V**	**Abr**	**Ba**	**Ca**	**Cp**	**La**	**Ma**	**Mo**	**Pu**	**To**	**Um**	**Sa**	**Si**
1.	*Actina chalybea* Meigen, 1804		•			•	•		•	•	•										
2.	*Adoxomyia dahlii* (Meigen, 1830)			•		•								•			•				•
3.	*Adoxomyia lindneri* Dušek & Rozkošný,1963			•										•							
4.	*Alliocera graeca* Saunders, 1845		•																		
5.	*Beris chalybata* (Forster, 1771)	•					•		•	•								•			
6.	*Beris clavipes* (Linnaeus, 1767)	•				•	•		•							•					
7.	*Beris fuscipes* Meigen, 1820					•				•											
8.	*Beris geniculata* Curtis, 1830					•	•			•											
9.	*Beris morrisii* Dale, 1841	•		•		•	•		•	•								•			
10.	*Beris strobli* Dušek & Rozkošný, 1968						•				•										
11.	*Beris vallata* (Forster, 1771)					•	•			•				•							
12.	*Chloromyia formosa* (Scopoli, 1763)	•	•	•	•	•	•	•	•	•	•	•	•	•		•	•	•	•	•	•
13.	*Chloromyia speciosa* (Macquart, 1834)		•			•	•			•	•			•							
14.	*Chorisops caroli* Troiano, 1995			•								•		•				•		•	
15.	*Chorisops masoni* Troiano & Toscano, 1995			•	•									•						•	•
16.	*Chorisops nagatomii* Rozkošný, 1979	•			•	•				•		•		•				•			
17.	*Chorisops tibialis* (Meigen, 1820)				•		•		•					•	•						
18.	*Chorisops tunisiae* (Becker, 1915)																			•	
19.	*Clitellaria ephippium* (Fabricius, 1775)	•	•		•	•	•	•	•	•				•	•			•	•		
20.	*Eupachygaster tarsalis* (Zetterstedt, 1842)	•				•			•					•				•			•
21.	*Hermetia illucens* (Linnaeus, 1758)			•	•	•			•		•	•		•			•	•		•	
22.	*Lasiopa calva* (Meigen, 1822)																				•
23.	*Lasiopa krkensis* Lindner, 1938		•																		
24.	*Lasiopa pseudovillosa* Rozkošný, 1983	•								•	•	•		•	•		•	•		•	•
25.	*Lasiopa tsacasi* Dušek & Rozkošný, 1970					•				•	•			•							
26.	*Lasiopa villosa* (Fabricius, 1794)	•	•	•		•				•				•			•				•
27.	*Microchrysa flavicornis* (Meigen, 1822)					•	•							•				•			
28.	*Microchrysa polita* (Linnaeus, 1822)					•	•		•					•				•		•	
29.	*Nemotelus (Camptopelta) nigrinus* Fallén, 1817																				
30.	*Nemotelus (Nemotelus) anchora* Loew, 1846	•																		•	
31.	*Nemotelus (Nemotelus) argentifer* Loew, 1846										•						•				
32.	*Nemotelus (Nemotelus) crenatus* Egger, 1859	•							•												
33.	*Nemotelus (Nemotelus) cylindricornis* Rozkošný, 1977																				•
34.	*Nemotelus (Nemotelus) lasiops* Loew, 1846																			•	•
35.	*Nemotelus (Nemotelus) latiusculus* Loew, 1871	•							•						•			•	•		
36.	*Nemotelus (Nemotelus) longirostris* Wiedemann, 1824																				•
37.	*Nemotelus (Nemotelus) maculiventris* Bigot, 1861																				•
38.	*Nemotelus (Nemotelus) nigrifrons* Loew, 1846																			•	•
39.	*Nemotelus (Nemotelus) niloticus* Olivier, 1811																			•	
40.	*Nemotelus (Nemotelus) notatus* Zetterstedt, 1842	•							•					•			•			•	
41.	*Nemotelus (Nemotelus) pantherinus* (Linnaeus, 1758)	•	•	•		•	•			•		•		•			•	•		•	
42.	*Nemotelus (Nemotelus) proboscideus* Loew, 1846																				•
43.	*Neopachygaster meromelas* (Dufour, 1841)													•							
44.	*Odontomyia angulata* (Panzer, 1798)	•	•		•	•			•				•	•			•	•		•	•
45.	*Odontomyia annulata* (Meigen, 1822)	•	•							•			•	•	•					•	
46.	*Odontomyia argentata* (Fabricius, 1794)						•														
47.	*Odontomyia cephalonica* Strobl, 1898																				•
48.	*Odontomyia discolor* Loew, 1846													•				•		•	•
49.	*Odontomyia flavissima* (Rossi, 1790)	•										**•**		•	•				•		•
50.	*Odontomyia hydroleon* (Linnaeus, 1758)	•	•		•	•	•	•	•	•				•				•			
51.	*Odontomyia ornata* (Meigen, 1822)	•			•	•			•					•				•		•	•
52.	*Odontomyia tigrina* (Fabricius, 1775)	•		•	•				•												
53.	*Oplodontha viridula* (Fabricius, 1775)	•	•		•	•	•	•	•	•				•			•	•		•	
54.	*Oxycera analis* Wiedemann *in* Meigen, 1822					•			•	•									•		
55.	*Oxycera fallenii* Stæger, 1844																				
56.	*Oxycera germanica* (Szilády, 1932)			•			•											•			
57.	*Oxycera leonina* (Panzer, 1798)		•	•	•	•	•		•									•			
58.	*Oxycera locuples* Loew, 1857				•	•	•	•										•			
59.	*Oxycera marginata* Loew, 1859											•		•							•
60.	*Oxycera meigenii* Stæger, 1844	•		•		•	•			•											
61.	*Oxycera morrisii* Curtis, 1833		•				•							•							•
62.	*Oxycera muscaria* (Fabricius, 1794)						•														
63.	*Oxycera nigricornis* Olivier, 1812	•			•	•	•		•	•				•			•			•	
64.	*Oxycera pardalina* Meigen, 1822	•		•		•	•	•	•					•				•			
65.	*Oxycera pseudoamoena* Dušek & Rozkošný, 1974			•			•														
66.	*Oxycera pygmaea* (Fallén, 1817)				•	•						•									
67.	*Oxycera rara* (Scopoli, 1763)	•		•		•	•											•		•	
68.	*Oxycera terminata* Meigen, 1822	•		•					•												
69.	*Oxycera trilineata* (Linnaeus, 1767)																			•	
70.	*Oxycera varipes* Loew in Heyden, 1870			•			•	•													
71.	*Pachygaster atra* (Panzer, 1798)	•	•	•	•	•	•		•	•				•	•			•	•		•
72.	*Pachygaster leachii* Curtis, 1824			•	•				•	•				•				•		•	
73.	*Sargus albibarbus* Loew, 1855			•					•			•		•							
74.	*Sargus bipunctatus* (Scopoli, 1763)	•	•	•	•	•			•	•				•						•	•
75.	*Sargus cuprarius* (Linnaeus, 1758)	•		•	•		•			•				•							•
76.	*Sargus flavipes* Meigen, 1822	•		•	•	•	•		•	•		•		•				•			
77.	*Sargus harderseni* Mason & Rozkošný, 2008				•				•												
78.	*Sargus iridatus* (Scopoli, 1763)	•		•	•	•	•	•		•			•	•	•		•				•
79.	*Sargus rufipes* Wahlberg, 1854					•	•					•									
80.	*Stratiomys cenisia* Meigen, 1822	•		•	•	•		•		•			•	•	•		•				•
81.	*Stratiomys chamaeleon* (Linnaeus, 1758)			•	•	•	•	•		•				•						•	
82.	*Stratiomys concinna* Meigen, 1822					•	•							•							
83.	*Stratiomys equestris* Meigen, 1835						•			•											
84.	*Stratiomys hispanica* (Pleske, 1901)																				•
85.	*Stratiomys longicornis* (Scopoli, 1763)	•	•							•		•	•	•						•	•
86.	*Stratiomys potamida* Meigen, 1822	•	•	•	•		•		•	•				•							
87.	*Stratiomys rubricornis* (Bezzi, 1896)	•								•	•				•						
88.	*Stratiomys singularior* (Harris, 1776)	•	•	•					•								•				•
89.	*Vanoyia tenuicornis* (Macquart, 1834)													•							
90.	*Zabrachia minutissima* (Zetterstedt, 1838)	**•**					•		**•**												
91.	*Zabrachia tenella* (Jaennicke, 1866)						•													•	
**Total**		**37**	**19**	**29**	**25**	**38**	**39**	**10**	**32**	**32**	**9**	**14**	**6**	**44**	**10**	**2**	**14**	**26**	**6**	**26**	**28**

## Short notes on the species new to the Italian fauna

### *Neopachygaster meromelas* (Dufour, 1841)

The larva of *Neopachygaster meromelas* has been described in detail by Rozkošný (1983) and by Stubbs and Drake (2001). The material examined was collected in Latium, Roma at “Tenuta della Cervelletta” 41°54'41.55"N, 12°34'57.15"E. Nine larvae were collected on 7.ii.2005 under decaying bark of a trunk of *Populus* sp. partially submerged in water; 3 ♂♂ and 6 ♀♀ emerged from reared larvae on v–vi/2005, M. Mei leg. (FMCV). *Neopachygaster meromelas* is a European species, known from Fennoscandia to the Pyrenees and North Caucasus (Rozkošný 1983), and has been recorded from the following countries: Belarus, Belgium, British Islands, Corsica, Czech Republic, Finland, France (mainland), Germany, Hungary, Poland, Russia (North and Northwest), Slovakia, Spain (mainland), Sweden (Rozkošný 2012) and Italy (this paper). In Italy *Neopachygaster meromelas* is known only in central Italy at “Tenuta della Cervelletta”, a small natural area (about 44 ha) located in the Northeast suburbs of Rome which is a relict wetland (Mason and Mei 2002). This site represents the southernmost European record of the species (cf. Rozkošný 1983).

**Figure 47.**
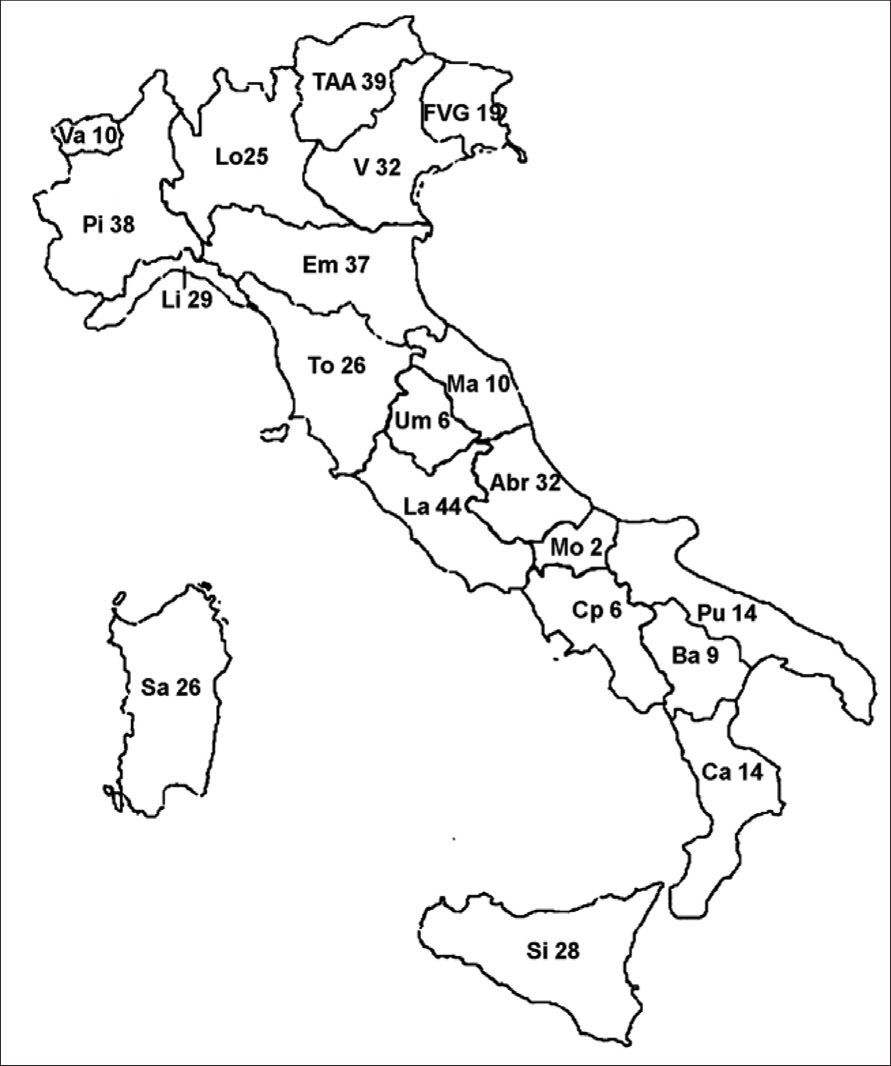
Number of species of the Stratiomyidae in the Italian administrative regions. N = Northern Italy: Em = Emilia-Romagna, FVG = Friuli-Venezia Giulia, Li = Liguria, Lo = Lombardy, Pi = Piedmont, TAA = Trentino-Alto Adige, V = Venetia, Va = Val Val d'Aosta. S = Peninsular Italy: Abr = Abruzzo, Ba = Basilicata, Ca = Calabria, Cp = Campania, La = Latium, Ma = Marches, Mo = Molise, Pu = Apulia, To = Tuscany, Um = Umbria. Si = Sicily and small circum-Sicilian islands. Sa = Sardinia and small circum-Sardinian islands.

### *Zabrachia minutissima* (Zetterstedt, 1838)

Venetia Region: Rovigo province, Porto Caleri, loc. Bosco Giardino, 45°05'N, 12°19'E, 12.viii–8.ix.2004, Malaise Trap, 2 ♀♀, D. Sommaggio leg. (FMCV); Emilia–Romagna, Ferrara province, Isola Bianca, LIPU Oasi, Retro Duna, 44°53'N, 11°38'E, 4.vii–1.viii.2004, Malaise Trap, 1 ♀, D. Sommaggio leg. (FMCV); Rovigo province, Porto Caleri, Bosco intermedio, 45°06'N, 12°19'E, 8.ix–1.x.2004, Malaise Trap, 1 ♀, D. Sommaggio leg. (FMCV); same data, but 20.vii–12.vii.2004, 1 ♀, (FMCV). Regione Veneto, Belluno province, Cellarda, Vincheto di Cellarda [State Nature Reserve], 230 m, UTM Latitude: 46°0'43"N, 11°58'32"E, 1–15.viii.2007, Window Trap T5 (cf. Audisio et al. 2008), G. Gatti & M. Dal Cortivo leg. (FMCV).

**Distribution.** *Zabrachia minutissima* is a Eurasian species (Rozkošný 1983): Czech Republic, Denmark (mainland), Finland, France (mainland), Germany, Greece (mainland), Hungary, Norway (mainland), Poland, Russia, Spain (mainland), Sweden, Switzerland, Ukraine, East Palaeartic and Near East (Rozkošný 2012).

### Other records new to the regions

*Hermetia illucens* is new to Calabria (Reggio Calabria, Pellaro, 19.ix.2011, photo by Francesco D’Aleo (2012)). *Stratiomys cenisia* is new to Sicily (Trapani 20.v.2009), and *Clitellaria ephippium* is new to Marche (12.vii.2010, photo by Marco Paglialunga). All these data were posted in the “Forum Entomologi italiani” [Forum of Italian Entomologists] www.entomologiitaliani.net/forum (last accessed 16 April 2012).

### Notes and key to European species of *Chorisops* Rondani, 1856: 173

The nomenclatorial history of the name *Chorisops* Rondani has been recently clarified by O’Hara et al. (2011).

In Europe, at the present time, five species of *Chorisops* are known: *Chorisops caroli* Troiano, 1995, *Chorisops masoni* Troiano & Toscano, 1995, *Chorisops nagatomii* Rozkošný, 1979, *Chorisops tibialis* (Meigen, 1820) and *Chorisops tunisiae* (Becker, 1915). Two of these, *Chorisops caroli* and *Chorisops masoni*, are probably endemic to Italy (Troiano 1995; Mason et al. 2006; Mason et al. 2009) (Figs 2–6).

**Figures 1–6.**
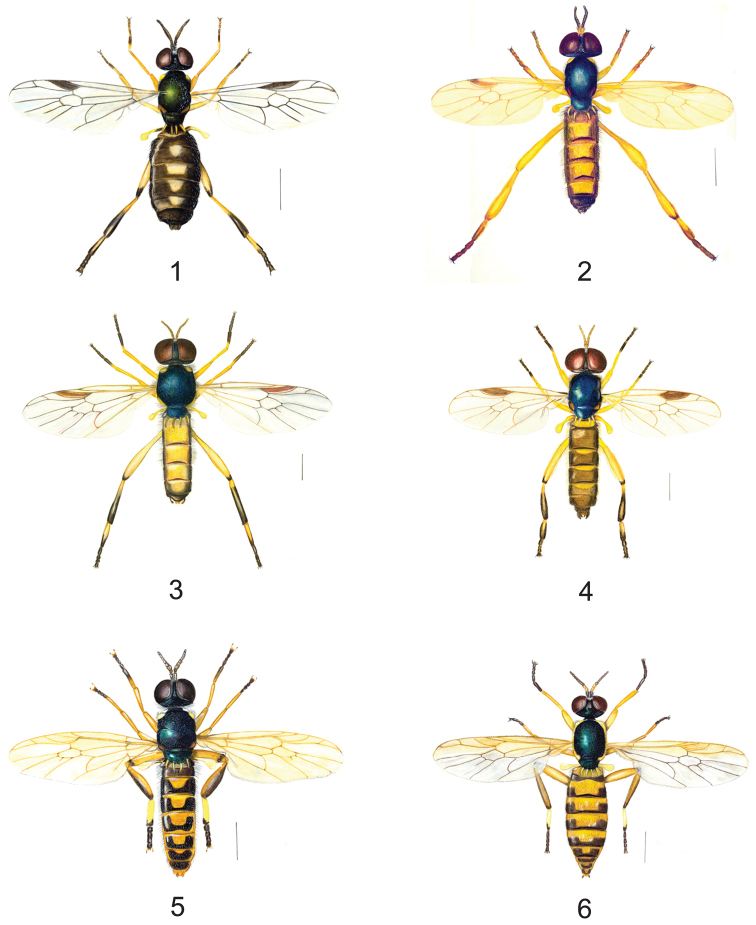
*Chorisops*, habitus: **1** *Chorisops caroli* Troiano, 1995 ♂ **2** *Chorisops masoni* Troiano & Toscano, 1995 ♂ **3** *Chorisops nagatomii* Rozkošný, 1979 ♂ **4** *Chorisops tibialis* (Meigen, 1820) ♂ **5** *Chorisops tunisiae* (Becker, 1915) ♂ **6** *Chorisops tunisiae* ♀, (drawns by Mason F). Scale bar = 1 mm.

**Figures 7–10.**
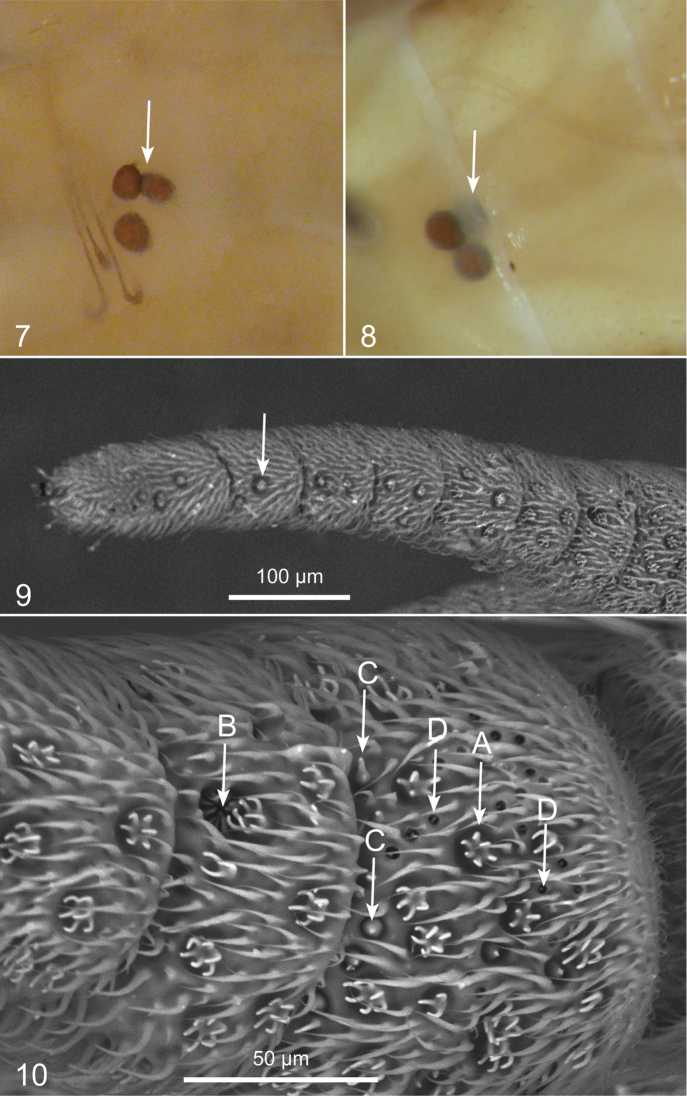
**7** Spermathecae of *Chorisops tunisiae* (Becker)**8** Spermathecae of *Chorisops tibialis* (Meigen) **9** Antenna of *Chorisops tunisiae* (Becker) **10** External side of the first (basal) flagellomere of *Chorisops tunisiae* (Becker). Antennal sensilla: **A** finger-like **B** sunken finger-like **C** subconical **D** stick-like.

As in other Beridinae (Woodley 2001) three subspherical spermathecae are present in the females of *Chorisops* (Figs 7, 8). The sensory pits on the external side of the first flagellomere, are up to four different types: finger-like (A), sunken finger-like in a pit (B), subconical (C) and stick-like inside a pit (D) (cf. Figs 9, 10). The males of *Chorisops nagatomii* (cf. also Stubbs and Drake 2001), were observed in a swarm over a shrub in a grassland and on flowers of *Hedera helix* L., in a floodplain forest (D. Birtele, pers. comm. 2012). In Italy, the peak of the flight period of *Chorisops nagatomii* and *Chorisops masoni* is generally between the second half of August and the first half of September (cf. Mason 2004), about one month later than the flight period of *Chorisops tibialis*. A new record is here reported for Piedmont for *Chorisops nagatomii*: 1 ♂ Alessandria province, Piovera, 44°57'43"N, 8°44'5"E, x.1933, G. C. Doria (in MCSNG).

### Key to the European species of *Chorisops*

**Figures 11–20.**
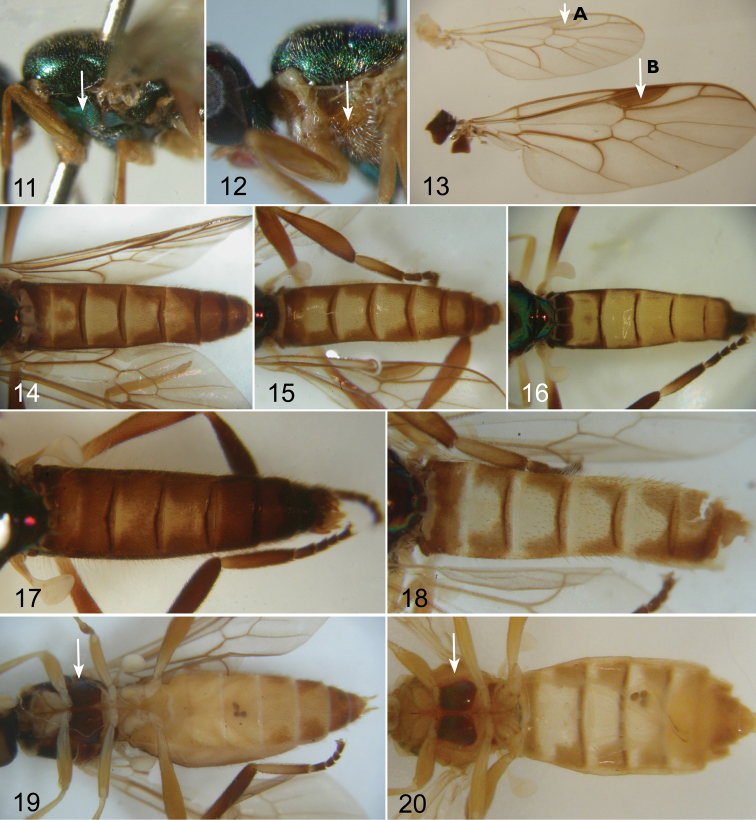
**11** Thorax in lateral view of *Chorisops nagatomii* Rozkošný **12** Thorax in lateral view of *Chorisops tunisiae* (Becker) **13** Wing pterostigma: **A** *Chorisops tunisiae* (Becker) **B** *Chorisops tibialis* (Meigen) **14–18** Male abdomen (dorsal view) of: **14** *Chorisops caroli* Troiano **15** *Chorisops masoni* Troiano & Toscano **16** *Chorisops nagatomii* Rozkošný **17** *Chorisops tibialis* (Meigen) **18** *Chorisops tunisiae* (Becker) **19–20** Male abdomen (ventral view) of: **19** *Chorisops tibialis* (Meigen) **20** *Chorisops tunisiae* (Becker).

Despite the availability of a relatively large amount of newly collected material of *Chorisops*, I have not been able to find any reliable external character of diagnostic value, except for the different colouring of the anepisternum and postpronotal callus (cf. Figs 11 and 12) and in the relative darkening of the wing pterostigma (Figs 13A, 13B). A reliable identification is possible only by examining the genitalia of both sexes.

### Key to males

**Table:** 

1	Pterostigma light yellow (Fig. 13A)	*Chorisops tunisiae* (Becker)
–	Pterostigma usually darker (Fig. 13B)	2
2	Abdominal tergites mainly brown (Figs 5, 17)	*Chorisops tibialis* (Meigen)
–	Tergites with more extensive yellow pattern (Figs 2, 3, 4, 6)	3
3	Tergites with only a narrow brown preapical grooves (Figs 4, 16); genitalia as in Figs 28-31	*Chorisops nagatomii* Rozkošný
–	Tergites with different colour pattern (Figs 2, 3, 14, 15)	4
4	Scutum shining green; genitalia as in Figs 25–27	*Chorisops masoni* Troiano & Toscano
–	Scutum shining blue; genitalia as in Figs 21–24	*Chorisops caroli* Troiano

**Figures 21–31.**
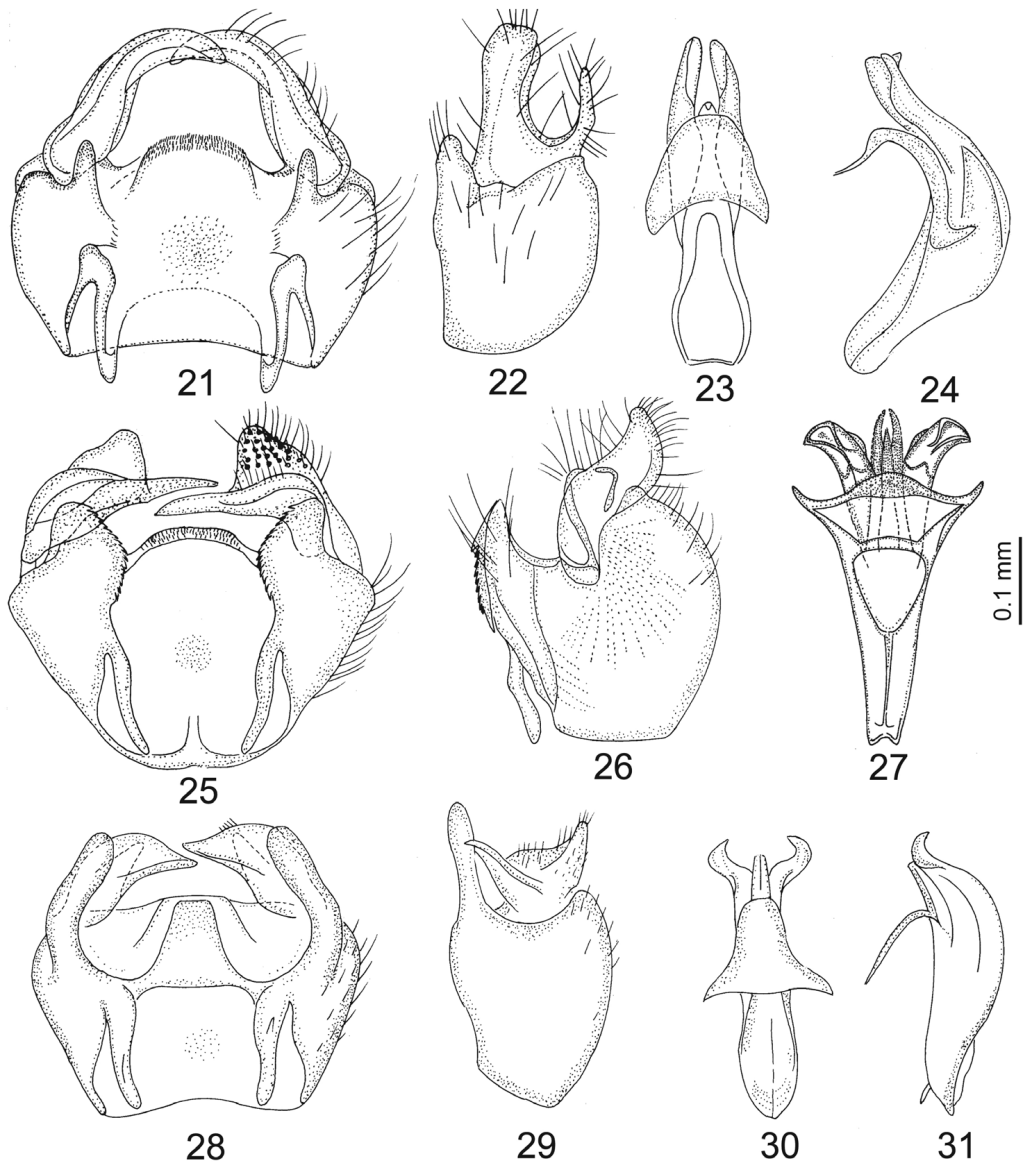
*Chorisops caroli*: **21** Genital capsule **22** Genital capsule (lateral view)**23** Aedeagal complex **24** Aedeagal complex (lateral view) **25–27** *Chorisops masoni*: **25** Genital capsule **26** Genital capsule (lateral view) **27** Aedeagal complex (ventral view) **28–31** *Chorisops nagatomii*: **28** Genital capsule **29** Genital capsule (lateral view) **30** Aedeagal complex (ventral view) **31** Aedeagal complex (lateral view); (redrawn from Rozkošný (1982) Troiano (1995) and Troiano and Toscano (1995).

### Key to females

**Table:** 

1	Pterostigma light yellow (Fig. 13A), anepisternum and postronotal callus yellow (Fig. 12), pleural sclerites bright yellow, except for the contrastingly black katepisternum (Fig. 20)	*Chorisops tunisiae* (Becker)
–	Pterostigma darker (Fig. 13B), anepisternum shining green (Fig. 11), pleural sclerites always dark (Fig. 19)	2
2	Genital furca with rounded corners (Figs 40, 42)	3
–	Genital furca with pointed corners (Figs 41, 43)	4
3	Genital furca massive, laterally enlarged, with a rounded median aperture (Fig. 40)	*Chorisops caroli* Troiano
–	Genital furca with a relatively wide transverse median aperture (Fig. 42)	*Chorisops nagatomii* Rozkošný
4	Genital furca with developed lateral wings (Fig. 41)	*Chorisops masoni* Troiano
–	Genital furca without developed lateral wings (Fig. 43)	*Chorisops tibialis* (Meigen)

**Figures 32–45.**
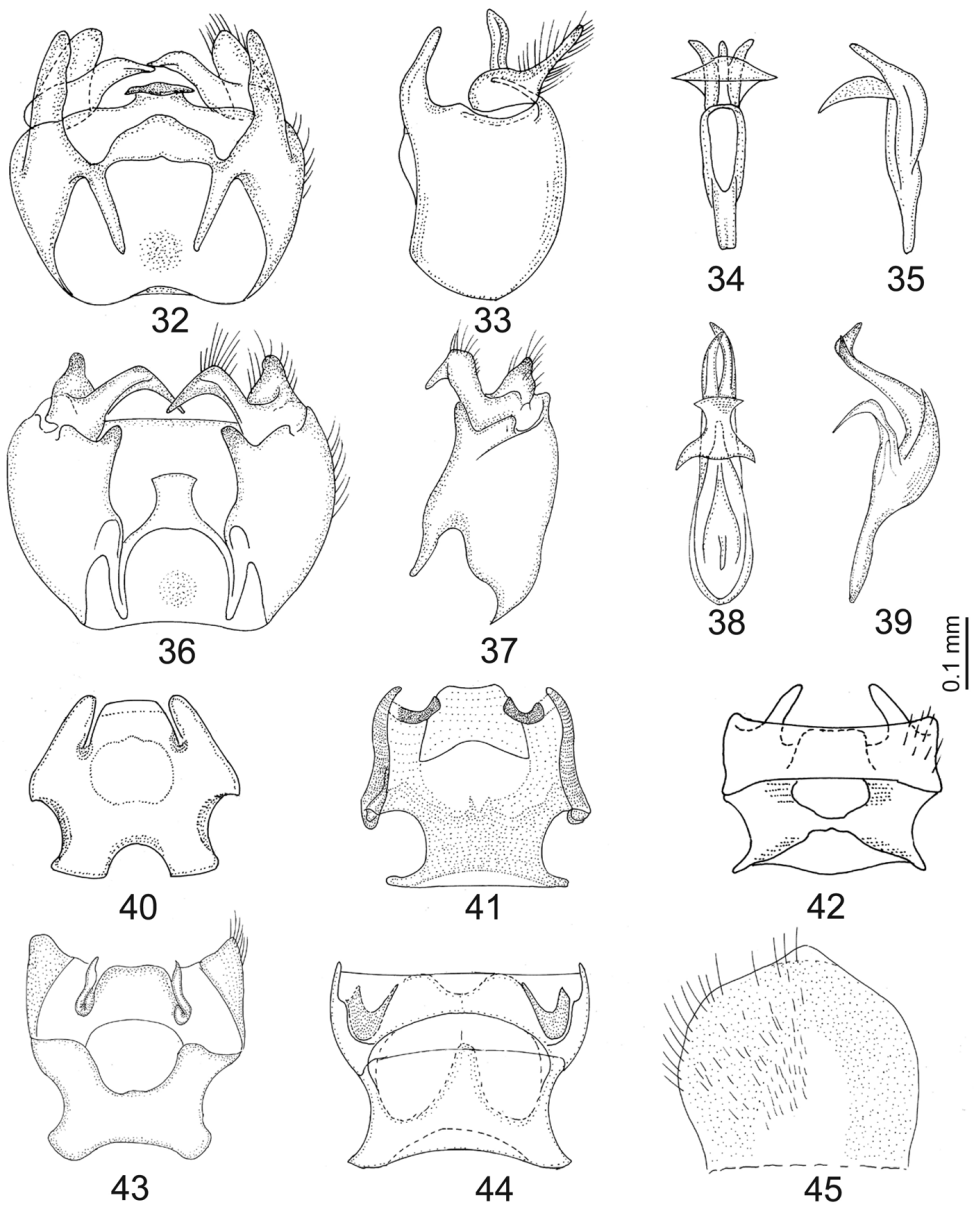
*Chorisops tibialis*: **32** Genital capsule **33** Genital capsule (lateral view) **34** Aedeagal complex **35** Aedeagal complex (lateral view) **36–39** *Chorisops tunisiae*: **36** Genital capsule **37** Genital capsule (lateral lateral view) **38** Aedeagal complex **39** Aedeagal complex (lateral view) **40–44** Genital furca of: **40** *Chorisops caroli* **41** *Chorisops masoni* **42** *Chorisops nagatomii* **43** *Chorisops tibialis* **44** *Chorisops tunisiae* **45** Sugenital plate of *Chorisops tunisiae*; (redrawn from Rozkošný (1982) Troiano (1995) and Troiano and Toscano (1995).

### Short faunistic notes

**Figure 46.**
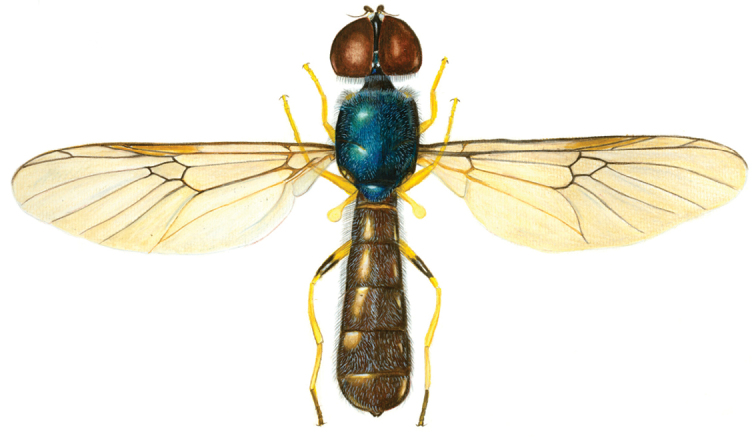
Habitus of *Sargus harderseni* Mason & Rozkošný, 2008 (♂), (drawn by Mason F).

With newly recorded *Eupachygaster meromelas* and *Zabrachia minutissima*, the Italian fauna includes at the present time 91 species. The species probably endemic to Italy are: *Chorisops caroli*, *Chorisops masoni* and *Sargus harderseni* (Fig. 46), the last recently described (Mason and Rozkošný 2008). The unique Italian record of *Vanoya tenuicornis* (Macquart, 1834), (Mason and Mei 2002) represents the southernmost European distribution of this species. The different regional distributions (cf. Tab. 1, Fig. 1), are evidently dependent on the intensity of the faunistic investigations. From the point of view of conservation, in Italy the most threatened species of soldier flies are those that have larvae which live in springs and in coastal salt marshes, because of water pollution and the progressive fragmentation and destruction of such habitats. Their conservation should start with (cf. Rozkošný 2005) building a European red list of endangered species, according to the IUCN categories (IUCN 2008; Farkač et al. 2005) as recently achieved for the saproxylic Coleoptera (Nieto and Alexander 2010).

## Appendix

Diptera Stratiomyidae, identified by Mason F. 2012 and 2013. (doi: 10.3897/zookeys.336.6016.app) File format: Microsoft Excel file (xls).

**Explanation note:** Diptera Stratiomyidae collected by Malaise trap in the framework of the Project LIFE_09_ENV_IT_0000078.
